# Supplementary material for: Association of iron homeostasis-related gene polymorphisms with pregnancy and neonatal outcomes in patients with gestational diabetes mellitus
Source: PLoS One. 2024 Dec 12;19(12):e0312180. doi: 10.1371/journal.pone.0312180 (PMC11637353; doi:10.1371/journal.pone.0312180)
Supplement: S1 Fig — A1 and B1:Mass spectrometry peak spectrum;A2 and B2:Mass spectrometry clustering diagram. (PDF) [file pone.0312180.s001.pdf]

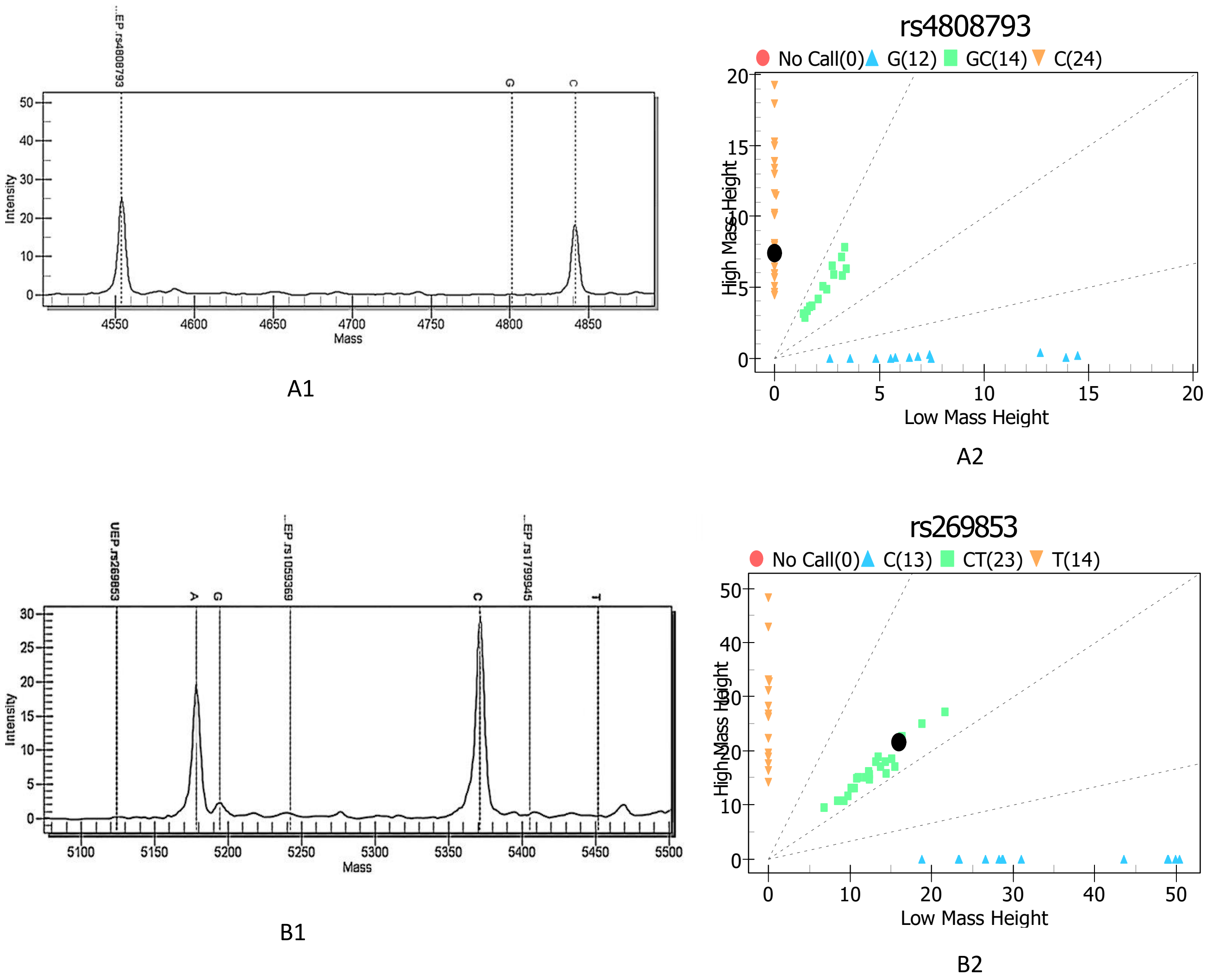

S1 fig: Mass spectrometry result chart. A1 and B1:Mass spectrometry peak spectrum;A2 and B2:Mass spectrometry clustering diagram.
