# Supplementary material for: Association of iron homeostasis-related gene polymorphisms with pregnancy and neonatal outcomes in patients with gestational diabetes mellitus
Source: PLoS One. 2024 Dec 12;19(12):e0312180. doi: 10.1371/journal.pone.0312180 (PMC11637353; doi:10.1371/journal.pone.0312180)
Supplement: S1 Table — (DOCX) [file pone.0312180.s002.docx]

S1Table: Gene polymorphism amplification primer sequence

| Gene | Variant | HGVS | Upstream amplification primers sequence | Downstream amplification primers sequence | Extended primer sequence | MAF |
| --- | --- | --- | --- | --- | --- | --- |
| GDF15 | rs4808793  (G>C) | [NC_000019.10:g.18383027G>C](https://www.ncbi.nlm.nih.gov/projects/sviewer/?id=NC_000019.10&search=NC_000019.10:g.18383027G>C&v=1:100&content=5), | ACGTTGGATGTTGTGCCTATGTCCAGCTTC | ACGTTGGATGTGCCTGTCACATGCAGACAC | CCTGTCCGGCAGATA | 0.63 |
| TMPRSS6 | rs855791  (A>G) | [NC_000022.11:g.37066896A>G](https://www.ncbi.nlm.nih.gov/projects/sviewer/?id=NC_000022.11&search=NC_000022.11:g.37066896A>G&v=1:100&content=5) | ACGTTGGATGATCCTTCTTGCCCTTGCGGT | ACGTTGGATGATGTGCAGTTGATCCCACAG | CACCTGGTAGCGATAG | 0.56 |
| C282Y | rs269853  (T>C) | [NC_000005.10:g.96471275T>C](https://www.ncbi.nlm.nih.gov/projects/sviewer/?id=NC_000005.10&search=NC_000005.10:g.96471275T>C&v=1:100&content=5) | ACGTTGGATGTCAGATATCAGGTGACAGAG | ACGTTGGATGTATGGTAATCTCAGTGGGGC | ACACTGAAGCCATACCT | 0.44 |
| GDF15 | rs1059369  (T>A) | NC_000019.10:g.18386331T>A | ACGTTGGATGTGGTTAGCAGGTCCTCGTAG | ACGTTGGATGAAGTTTCCCGGGACCCTCA | GAATCTGGAGTCTTCGG | 0.22 |
| H63D | rs1799945  (C>G) | NC_000006.12:g.26090951C>G | ACGTTGGATGTGGAAACCCATGGAGTTCGG | ACGTTGGATGGTTTGAAGCTTTGGGCTACG | CCACACGGCGACTCTCAT | 0.14 |
| BMP2 | rs173107  (A>C) | NC_000020.11:g.6785194A>C | ACGTTGGATGCTGTGTACACATGCAAGATG | ACGTTGGATGCGGTAAGATGTTTCAGGAGC | ACACATGTCTAACTTGGCA | 0.64 |
| C282Y | rs3811647  (G>A) | NC_000003.12:g.133765185G>A | ACGTTGGATGCCTAAGCTGAGGGAGTTTAC | ACGTTGGATGTGAGGATCAGTGGGAAAGAC | tGGGAGTTTACAGACAGATC | 0.33 |
| CUBN | rs10904850  (G>A) | NC_000010.11:g.16955708G>A | ACGTTGGATGTGCCTCTGTTGTCCTGTTCT | ACGTTGGATGATGTGCCAGGAACTGGATTC | ACGTTGGATGATGTGCCAGGAACTGGATTC | 0.27 |
| FADS2 | rs174577  (C>A) | NC_000011.10:g.61837342C>A | ACGTTGGATGGGTAGTATTCGGTCTTGTCC | ACGTTGGATGACCACCCACCATCTCTTACT | GGTCTTGTCCTTTTCATTGAC | 0.35 |
| TF | rs8177240  (T>G) | NC_000003.12:g.133758857T>C | ACGTTGGATGCTGTGTTGGAGCTTCTGTTC | ACGTTGGATGCTTAACTACTGGAGGTGTTC | CCTGACAATAAACAATGAACA | 0.31 |
| TFR2 | rs7385804  (C>A) | NC_000007.14:g.100638347C>A | ACGTTGGATGCAAACTACTGGCCTCAAAC | ACGTTGGATGAAAAGCCCTGAGCAGGCTGG | AAATGCTGGGATTATAGGAAT | 0.64 |
| C282Y | rs1800562  (G>A) | NC_000006.12:g.26092913G>A | ACGTTGGATGTACCCCAGATCACAATGAGG | ACGTTGGATGTGGATAACCTTGGCTGTACC | aaggaCTGGGTGCTCCACCTGG | 0.053 |
