# Supplementary material for: Association of iron homeostasis-related gene polymorphisms with pregnancy and neonatal outcomes in patients with gestational diabetes mellitus
Source: PLoS One. 2024 Dec 12;19(12):e0312180. doi: 10.1371/journal.pone.0312180 (PMC11637353; doi:10.1371/journal.pone.0312180)
Supplement: S2 Table — (DOCX) [file pone.0312180.s003.docx]

|  | | S2 table: Association of gene phenotype with gestational diabetes mellitus | | | | | |
| --- | --- | --- | --- | --- | --- | --- | --- |
| gene | variant | Genotype/allele | Non-GDM（n=74) | GDM（n=138) | p | P for FDR | Crude OR (95% CI) |
| GDF15 | rs1059369 | TT | 31 | 54 |  |  |  |
|  |  | AT | 37 | 58 | 0.732 | 0.934 | 1.111(0.607-2.033) |
|  |  | AA | 6 | 26 | 0.072 | 0.544 | 0.634(0.386-1.041) |
|  |  | T | 99 | 166 |  |  |  |
|  |  | A | 49 | 110 | 0.171 | 0.739 | 0.747(0.491-1.135) |
| CUBN | rs10904850 | GG | 53 | 102 |  |  |  |
|  |  | AG | 20 | 30 | 0.456 | 0.883 | 1.283(0.666-2.472) |
|  |  | AA | 1 | 6 | 0.298 | 0.832 | 0.566(0.194-1.653) |
|  |  | G | 126 | 234 |  |  |  |
|  |  | A | 22 | 42 | 0.923 | 0.939 | 0.973(0.556-1.702) |
| BMP2 | rs173107 | AA | 34 | 66 |  |  |  |
|  |  | AC | 30 | 59 | 0.966 | 0.941 | 0.987(0.54-1.805) |
|  |  | CC | 10 | 13 | 0.394 | 0.867 | 1.222(0.77-1.938) |
|  |  | A | 98 | 191 |  |  |  |
|  |  | C | 54 | 85 | 0.529 | 0.898 | 1.146(0.749-1.755) |
| FADS2 | rs174577 | CC | 21 | 28 |  |  |  |
|  |  | AC | 32 | 70 | 0.168 | 0.736 | 0.61(0.302-1.232) |
|  |  | AA | 21 | 40 | 0.366 | 0.859 | 0.837(0.568-1.232) |
|  |  | C | 74 | 126 |  |  |  |
|  |  | A | 74 | 150 | 0.393 | 0.867 | 0.913(0.743-1.122) |
| H63D | rs1799945 | CC | 70 | 133 |  |  |  |
|  |  | CG | 4 | 5 | 0.542 | 0.900 | 1.52(0.396-5.841) |
|  |  | C | 144 | 271 |  |  |  |
|  |  | G | 4 | 5 | 0.544 | 0.900 | 1.506(0.398-5.694) |
| C282Y | rs269853 | TT | 16 | 38 |  |  |  |
|  |  | CT | 36 | 69 | 0.554 | 0.902 | 1.239(0.609-2.519) |
|  |  | CC | 22 | 31 | 0.201 | 0.769 | 1.298(0.87-1.937) |
|  |  | T | 68 | 145 |  |  |  |
|  |  | C | 80 | 131 | 0.196 | 0.765 | 1.302(0.873-1.943) |
| C282Y | rs3811647 | GG | 25 | 51 |  |  |  |
|  |  | AG | 39 | 65 | 0.524 | 0.897 | 1.224(0.657-2.279) |
|  |  | AA | 10 | 22 | 0.868 | 0.935 | 0.963(0.618-1.501) |
|  |  | G | 89 | 167 |  |  |  |
|  |  | A | 59 | 109 | 0.94 | 0.940 | 1.016(0.675-1.527) |
| GDF15 | rs4808793 | GG | 9 | 13 |  |  |  |
|  |  | CG | 43 | 60 | 0.942 | 0.940 | 1.035(0.406-2.639) |
|  |  | CC | 22 | 65 | 0.151 | 0.715 | 0.699(0.429-1.14) |
|  |  | G | 61 | 86 |  |  |  |
|  |  | C | 87 | 190 | 0.038* | 0.544 | 0.646(0.426-0.977) |
| TFR2 | rs7385804 | CC | 3 | 9 |  |  |  |
|  |  | AC | 26 | 37 | 0.296 | 0.831 | 2.108(0.52-8.545) |
|  |  | AA | 45 | 92 | 0.579 | 0.906 | 1.211(0.615-2.384) |
|  |  | C | 32 | 55 |  |  |  |
|  |  | A | 116 | 221 | 0.681 | 0.919 | 0.902(0.553-1.473) |
| TF | rs8177240 | TT | 25 | 49 |  |  |  |
|  |  | TG | 38 | 67 | 0.74 | 0.925 | 1.112(0.595-2.077) |
|  |  | GG | 11 | 22 | 0.964 | 0.940 | 0.99(0.641-1.529) |
|  |  | T | 88 | 165 |  |  |  |
|  |  | G | 60 | 111 | 0.948 | 0.940 | 1.014(0.675-1.522) |
| TMPRSS6 | rs855791 | AA | 42 | 73 |  |  |  |
|  |  | AG | 25 | 32 | 0.354 | 0.855 | 1.358(0.711-2.592) |
|  |  | GG | 7 | 33 | 0.03* | 0.544 | 0.607(0.387-0.952) |
|  |  | A | 109 | 178 |  |  |  |
|  |  | G | 39 | 98 | 0.055 | 0.544 | 0.65(0.418-1.01) |
| *p<0.05 | |  | | | | | |
