# Supplementary material for: Association of iron homeostasis-related gene polymorphisms with pregnancy and neonatal outcomes in patients with gestational diabetes mellitus
Source: PLoS One. 2024 Dec 12;19(12):e0312180. doi: 10.1371/journal.pone.0312180 (PMC11637353; doi:10.1371/journal.pone.0312180)
Supplement: S3 Table — (DOCX) [file pone.0312180.s004.docx]

**S3 table : Correlation of GDM hime gene polymorphisms with ferritin and hemoglobin levels**

|  |  |  |  | ferritin | | HB | |
| --- | --- | --- | --- | --- | --- | --- | --- |
| gene | variant | Genotype/  Allele | n | Ferritin  (Mid pregnancy ) | Ferritin  (Late pregnancy ) | HB  (Mid pregnancy ) | HB  (Late pregnancy ) |
| GDF15 | rs1059369 | TT | 54 | 27.7（18.48-47.48） | 27.3(20.23-41.78) | 116.86±1.35 | 122.43±1.33 |
|  |  | AT | 58 | 25.65（14.35-43.48） | 30.25(17.03-48.03 | 118.17±1.38 | 122.98±1.22 |
|  |  | AA | 26 | 27.9（18.03-56.43） | 32.25(17.1-50.75) | 113.96±1.13 | 118.15±1.57 |
| CUBN | rs10904850 | GG | 102 | 26.6（16.85-44.23） | 28.35(18.33-43.38) | 116.17±0.91 | 121.61±0.93 |
|  |  | AG | 30 | 28.25（16.75-53.13） | 27.8(16.95-47.33) | 119.17±2.05 | 122.17±1.60 |
|  |  | AA | 6 | 66.6（32.68-82.18） | 39.35(29.25-55.6) | 117.17±3.22 | 124.5±5.35 |
| BMP2 | rs173107 | AA | 66 | 23.5（17.08-51.28） | 27.75(17.15-40.75) | 115.08±1.11 | 120.33±1.026 |
|  |  | AC | 59 | 28.2（16.9-43.3） | 30.7(19.1-47.8) | 118.70±1.28 | 122.61±1.26^*^ |
|  |  | CC | 13 | 27（19.2-44） | 33.7(19.15-48.2) | 117.62±2.82 | 126.15±3.20 |
| FADS2 | rs174577 | CC | 28 | 28.55（17.38-47.03） | 36.35(22.28-47.43) | 116.79±2.10 | 123.25±1.80 |
|  |  | AC | 70 | 26.5（16.83-48.13） | 27.05(16.95-41.78) | 117.6±1.168 | 122.01±1.17 |
|  |  | AA | 40 | 27.45(17.05-41.7) | 29.25(20-49.68) | 115.6±1.30 | 120.6±1.34 |
| H63D | rs1799945 | CC | 133 | 27(17.15-44.45) | 28.7(18.7-44.4) | 116.96±0.84 | 121.94±0.82 |
|  |  | CG | 5 | 70.9(27.75-84.15) | 40.2(16.25-74.55) | 114.2±3.01 | 119.6±3.04 |
| C282Y | rs269853 | TT | 38 | 27.45(16.45-48.2) | 27.5(19.95-47.33) | 115.55±1.27 | 120.7105±1.16 |
|  |  | CT | 69 | 27.5(19.7-45.3) | 31.7(17.15-46.9) | 117.14±1.11 | 122.2319±1.21 |
|  |  | CC | 31 | 23.9(12.1-45) | 28(19.4-36.5) | 117.84±2.21 | 122.4194±1.85 |
| C282Y | rs3811647 | GG | 51 | 23.5（12.8-49.7） | 28(16.2-42.9) | 115.82±1.40 | 121.59±1.22 |
|  |  | AG | 65 | 27.6（18.6-45） | 29.8(18.7-48.9) | 118.3±1.112 | 122.91±1.137 |
|  |  | AA | 22 | 29.4（14.4-44.23） | 30.25(22.33-46.53) | 114.95±2.22 | 119.36±2.39 |
| GDF15 | rs4808793 | GG | 13 | 45(21.8-50.55) | 31.7(24.55-42.25) | 116±2.62 | 123.69±3.31 |
|  |  | CG | 60 | 26(17.75-44.5) | 28.8(19.55-46.78) | 116.92±1.29 | 122.07±1.22 |
|  |  | CC | 65 | 27.3(15.35-43.9) | 28.7(16.65-46.35) | 116.99±1.18 | 121.23±1.09 |
| TFR2 | rs7385804 | CC | 9 | 27.3(12.9-39.75) | 27(12.85-28.75) | 120.11±2.55 | 125.89±1.94 |
|  |  | AC | 37 | 31.6(21.55-50.3) | 35.5(18.9-51.15) | 116.68±1.62 | 120.70±1.34 |
|  |  | AA | 92 | 23.7(16.95-45) | 28.95(18.95-43.9) | 116.62±1.01 | 121.92±1.05 |
| TF | rs8177240 | TT | 49 | 23.5(12.75-51.6) | 28(16.65-43.85) | 115.71±1.45 | 121.71±1.26 |
|  |  | TG | 67 | 27.6(18.8-45) | 29.8(18.5-48.7) | 118.33±1.08 | 122.78±1.11 |
|  |  | GG | 22 | 29.4(14.4-44.23 | 30.25(22.33-46.5) | 114.96±2.22 | 119.36±2.39 |
| TMPRSS6 | rs855791 | AA | 73 | 25.7(18.3-44.95) | 28.7(15.95-41.25) | 116.22±1.11 | 120.41±1.137 |
|  |  | AG | 32 | 28.5(13.43-46.8) | 29.65(17.13-48.1) | 118.56±2.039 | 123.34±1.65 |
|  |  | GG | 33 | 27.8(13.55-55.55) | 28(20.9-51.85) | 116.64±1.34 | 123.60±1.44 |
| ^*^P<0.05 | | | | | | | |
