# Supplementary material for: Association of iron homeostasis-related gene polymorphisms with pregnancy and neonatal outcomes in patients with gestational diabetes mellitus
Source: PLoS One. 2024 Dec 12;19(12):e0312180. doi: 10.1371/journal.pone.0312180 (PMC11637353; doi:10.1371/journal.pone.0312180)
Supplement: Attachment — Submitted filename: Response to Reviewers.docx [file pone.0312180.s005.docx]

July11, 2024

PLOS ONE

Dear Editor:

Thank you for your letter and comments on our manuscript titled “Association of iron homeostasis gene polymorphisms with pregnancy and neonatal outcomes in patients with gestational diabetes mellitus”(ID:PONE-D-24-26003). These comments helps us improve our manuscript. We have read the editor’ comments carefully and marked the revised portions in red and highlighted them yellow in the manuscript.

**Responses to the Editor’ comments:**

**Comment:** We recognize that you obtained ethical approval from your local ethics committee after you began the study: your ethics approval document is dated July 6, 2023, while personal data were collected and accessed beginning on January 5th, 2023. For human subjects research, PLOS ONE requires that authors obtain ethics committee approval before beginning the study, and we do not accept retrospective ethical approval.

**Response :** Due to my mistake, I mistakenly wrote "September 5th" as "January 5th". The date when were accessed for research purposes is September 5th, 2024 (page5,line122-123), we obtained ethics committee approval before beginning the study. We believe that the submission meets the ethical requirements for human subjects research submissions.

We deeply appreciate your consideration of our manuscript, and we look forward to receiving comments from the reviewers. If you have any queries, please don’t hesitate to contact me at the address below.

Thank you and best regards.

Yours sincerely,

**Qichang Wu,** Director ,Center of Prenatal Diagnosis, Women and Children’s Hospital, School of Medicine, Xiamen University, China.

Mailing address: No 10 Zhenhai Road, Xiamen City, Fujian Province, China

Tel.: (86)592-2662071 GMT+8h

E-mail: qichang_wu@163.com.

**Huiming Ye PhD,** Director, Department of Laboratory Medicine, Fujian Key Clinical Specialty of Laboratory Medicine, Women and Children’s Hospital, School of Medicine, Xiamen University and Associate Professor of Laboratory Medicine, School of Public Health, Xiamen University.

Mailing address: No 10 Zhenhai Road, Xiamen City, Fujian Province, China

Telephone: (86)592-2662071 GMT+8h

E-mail: yehuiming@xmu.edu.cn
